# Concurrent Acute Appendicitis and Cholecystitis: A Systematic Literature Review

**DOI:** 10.3390/jcm14145019

**Published:** 2025-07-15

**Authors:** Adem Tuncer, Sami Akbulut, Emrah Sahin, Zeki Ogut, Ertugrul Karabulut

**Affiliations:** 1 Department of Surgery, Istanbul Aydin University Faculty of Medicine, 34295 Istanbul, Turkey; ademtuncer89@hotmail.com (A.T.);; 2 Department of Surgery and Liver Transplantation, Inonu University Faculty of Medicine, 44280 Malatya, Turkey; 3 Department of Surgery, Elazig Fethi Sekin City Hospital, 23280 Elazig, Turkey

**Keywords:** acute appendicitis, acute cholecystitis, concurrent diseases, synchronous diseases, simultaneous diseases, laparoscopy, diagnostic modalities

## Abstract

**Background**: This systematic review aimed to comprehensively evaluate the clinical, diagnostic, and therapeutic features of synchronous acute cholecystitis (AC) and acute appendicitis (AAP). **Methods**: The review protocol was prospectively registered in PROSPERO (CRD420251086131) and conducted in accordance with PRISMA 2020 guidelines. A systematic search was performed across PubMed, MEDLINE, Web of Science, Scopus, Google Scholar, and Google databases for studies published from January 1975 to May 2025. Search terms included variations of “synchronous,” “simultaneous,” “concurrent,” and “coexistence” combined with “appendicitis,” “appendectomy,” “cholecystitis,” and “cholecystectomy.” Reference lists of included studies were screened. Studies reporting human cases with sufficient patient-level clinical data were included. Data extraction and quality assessment were performed independently by pairs of reviewers, with discrepancies resolved through consensus. No meta-analysis was conducted due to the descriptive nature of the data. **Results**: A total of 44 articles were included in this review. Of these, thirty-four were available in full text, one was accessible only as an abstract, and one was a literature review, while eight articles were inaccessible. Clinical data from forty patients, including two from our own cases, were evaluated, with a median age of 41 years. The gender distribution was equal, with a median age of 50 years among male patients and 36 years among female patients. Leukocytosis was observed in 25 of 33 patients with available laboratory data. Among 37 patients with documented diagnostic methods, ultrasonography and computed tomography were the most frequently utilized modalities, followed by physical examination. Twenty-seven patients underwent laparoscopic cholecystectomy and appendectomy. The remaining patients were managed with open surgery or conservative treatment. Postoperative complications occurred in five patients, including sepsis, perforation, leakage, diarrhea, and wound infections. Histopathological analysis revealed AAP in 25 cases and AC in 14. Additional findings included gangrenous inflammation and neoplastic lesions. **Conclusions**: Synchronous AC and AAP are rare and diagnostically challenging conditions. Early recognition via imaging and clinical evaluation is critical. Laparoscopic management remains the preferred approach. Histopathological examination of surgical specimens is essential for identifying unexpected pathology, thereby guiding appropriate patient management.

## 1. Introduction

Acute appendicitis (AAP) remains one of the most common surgical emergencies worldwide and continues to constitute a major cause of acute abdominal pain that requires urgent intervention [1,2]. Since its first clinical characterization by Reginald Heber Fitz in 1886, AAP has remained one of the most frequently encountered surgical emergencies in clinical practice [3,4]. Clinically, it is characterized by abdominal pain that initially presents around the umbilicus and subsequently migrates to McBurney’s point in the right lower quadrant. Physical examination typically reveals localized tenderness, rebound pain, and abdominal rigidity, along with systemic symptoms such as fever, nausea, vomiting, and anorexia [5,6]. Obstruction of the appendiceal lumen by a fecalith or lymphatic hyperplasia is a primary trigger for the inflammatory process, leading to increased intraluminal pressure, bacterial overgrowth, and ultimately appendiceal ischemia and necrosis [7]. Epidemiologically, the lifetime risk of developing AAP is estimated at 8.6% in men and 6.7% in women, while population-based studies suggest that approximately 12% of men and 23% women will undergo appendectomy during their lifetime [8]. While classic AAP typically presents with well-recognized signs and symptoms, atypical or aberrantly positioned appendices can mimic other intra-abdominal pathologies, leading to diagnostic challenges and potential delays in appropriate management [9]. Because of these variations in clinical presentation, the diagnosis of AAP is usually based on a combination of medical history, physical examination findings, laboratory parameters, and radiological imaging results, such as ultrasound (US), computed tomography (CT), or magnetic resonance imaging (MRI), the latter being preferred during pregnancy [10]. Despite emerging interest in nonoperative management for selected uncomplicated cases, the surgical approach (appendectomy) remains the definitive treatment, particularly in complicated or advanced presentations where perforation, abscess formation, or peritonitis may develop [11,12]. Among the surgical techniques, laparoscopic appendectomy, pioneered by Kurt Semm in 1983 [13], marked a pivotal advancement in the minimally invasive management of AAP and has progressively supplanted open appendectomy. The advantages of laparoscopic appendectomy include reduced postoperative pain, shorter hospital stays, lower wound infection rates, and faster convalescence [13,14].

Acute cholecystitis (AC), another frequent etiology of acute abdomen, similarly requires prompt diagnosis and often urgent surgical intervention [15]. The management of cholecystitis has undergone fundamental transformation following the introduction of cholecystectomy, first successfully performed by Carl Langenbuch in 1882, establishing cholecystectomy as the definitive treatment for gallbladder diseases [16,17]. Clinically, cholecystitis is characterized by persistent right upper quadrant or epigastric pain, a positive Murphy’s sign, fever, nausea, and vomiting, often accompanied by leukocytosis and elevated acute-phase reactants [18,19]. In the vast majority of cases (90–95%), AC results from gallstone-induced obstruction of the cystic duct, which leads to gallbladder distension, secondary bacterial colonization, mucosal injury, and transmural inflammation [20,21]. Its incidence shows a predilection for females and increases progressively with advancing age, peaking between the fourth and sixth decades of life. In Western populations, the high prevalence of cholelithiasis creates a significant healthcare burden, with nearly 200,000 cases of AC diagnosed annually in the United States alone [20,22]. Hepatobiliary surgeons often recommend laparoscopic cholecystectomy, which was first successfully performed by Erich Mühe in 1985 [23]. The technique gained widespread recognition following Philippe Mouret’s work in 1987 [24], laying the foundation for its global adoption and representing a pivotal advancement in the minimally invasive surgical management of cholecystitis. For patients presenting within 72 h of symptom onset, early laparoscopic cholecystectomy is recommended. In patients with symptoms lasting longer than 72 h, initial conservative management with antibiotics is preferred, followed by delayed laparoscopic cholecystectomy within 4–8 weeks [25,26].

While AAP and AC are independently well-characterized clinical entities, their synchronous occurrence remains exceedingly rare and poorly understood, particularly regarding the underlying pathophysiological mechanisms, clinical presentation, and optimal management strategies. To date, a total of 44 articles have been published on this topic [5,15,19,27,28,29,30,31,32,33,34,35,36,37,38,39,40,41,42,43,44,45,46,47,48,49,50,51,52,53,54,55,56,57,58,59,60,61,62,63,64,65,66,67]. Among these, one study, authored by Buhamed et al. [30], presented a very brief narrative review based on 11 cases, while four other publications, such as those by Aljunaydil et al. [39], Kancheva et al. [51], Thompson et al. [64], and Gandhi et al. [47], described individual case reports each accompanied by concise literature analysis. However, no comprehensive systematic review has previously consolidated all available case reports and clinical data regarding the synchronous presentation of these two conditions.

The simultaneous presentation of these two pathologies poses significant diagnostic and therapeutic challenges, often due to overlapping clinical features that can obscure the clinical picture and confound timely decision making. The localization of pain may vary depending on the anatomical positioning of the inflamed appendix. In particular, variations in appendiceal positioning, such as a subhepatic appendix, further complicate the differential diagnosis [5]. Hypotheses have included hematogenous bacterial dissemination via the portal venous system, systemic inflammatory activation, or coincidental coexistence of two independent pathologies triggered by shared risk factors or microbial agents [47]. Regardless of the mechanism, clinicians must maintain heightened clinical suspicion in patients presenting with atypical or shifting abdominal symptoms, and diagnostic laparoscopy may serve as both a diagnostic and therapeutic modality when noninvasive imaging proves inconclusive.

In recent years, laparoscopic surgery has emerged as the favored technique for addressing synchronous AC and AAP, offering numerous advantages such as reduced postoperative morbidity, shorter hospitalization, expedited recovery, diminished infection rates, superior cosmesis, and improved overall patient satisfaction [68]. To address gaps in understanding the underlying pathophysiological mechanisms that might explain simultaneous inflammation, the diagnostic challenges due to overlapping clinical features that often delay or obscure timely identification, and the uncertainties surrounding the best surgical and medical management strategies for synchronous AC and AAP, this study undertakes a comprehensive systematic review of all previously published cases. Through this review, we aim to enrich the understanding of disease behavior, refine diagnostic pathways, support evidence-based surgical decision making, and highlight key topics that deserve further scientific investigation to improve patient outcomes in this rare clinical scenario.

## 2. Materials and Methods

### 2.1. Systematic Review

#### 2.1.1. Protocol Registration

This systematic review was prospectively registered in PROSPERO (CRD420251086131) 2 July 2025, and it was conducted in line with the Preferred Reporting Items for Systematic Reviews and Meta-Analyses (PRISMA) 2020 guidelines to ensure methodological rigor and transparency [69]. A PRISMA flow diagram was prepared to illustrate the process of study identification, screening, eligibility, and inclusion.

#### 2.1.2. Literature Search Strategy

A comprehensive search was conducted across PubMed, Scopus, Web of Science, Google Scholar, and Google for publications from January 1975 to May 2025. Search terms included variations of simultaneous, synchronous, concurrent, or coexisting appendicitis and cholecystitis. Reference lists of all the included studies were manually reviewed. No restrictions were applied regarding language, country, or publication type. References were managed using EndNote 21.5 (Bld 18513). Search strings included the following: PubMed: (simultaneous[Title/Abstract] OR synchronous[Title/Abstract] OR concurrent[Title/Abstract] OR coexistence[Title/Abstract]) AND (appendicitis[Title/Abstract] OR appendectomy[Title/Abstract]) AND (cholecystitis[Title/Abstract] OR cholecystectomy[Title/Abstract]). Web of Science: Topic Search=(simultaneous OR synchronous OR concurrent OR coexistence) AND S=(appendicitis OR appendectomy) AND (cholecystitis OR cholecystectomy). Scopus: TITLE-ABS-KEY (simultaneous OR synchronous OR concurrent OR coexistence) AND (appendicitis OR appendectomy) AND (cholecystitis OR cholecystectomy). Google Scholar and Google: (“simultaneous” OR “synchronous” OR “concurrent” OR “coexistence”) AND (“appendicitis” OR “appendectomy”) AND (“cholecystitis” OR “cholecystectomy”). Approximately the first 20 pages of Google and Google Scholar results were screened. Boolean operators (AND/OR) were utilized to optimize the search sensitivity [70]. Reference lists of eligible studies were manually reviewed to identify additional relevant publications. No language, country, or publication type restrictions were applied.

#### 2.1.3. Eligibility Criteria

Inclusion criteria comprised studies reporting human cases of synchronous AC and AAP with adequate clinical, diagnostic, therapeutic, and outcome data. Studies were excluded if they lacked patient-level data, involved non-human subjects, were purely editorial or commentary articles, or were inaccessible despite exhaustive searching.

#### 2.1.4. Study Selection and Data Extraction

Pairs of independent reviewers screened titles, abstracts, and full texts for eligibility. Data extraction was also performed independently in pairs using a standardized form. In cases of disagreement, joint meetings were held among reviewer pairs to reach consensus. Extracted variables included first author name, publication year, country of origin, language, study type, patient demographics (age, sex), presenting symptoms (pain characteristics, fever, nausea, vomiting, anorexia), laboratory results (white blood cell counts), imaging modalities utilized [ultrasonography (US), computed tomography (CT), magnetic resonance imaging (MRI)], preoperative diagnosis, intraoperative findings, surgical approaches, histopathological results of both appendix and gallbladder specimens, postoperative complications, hospital stay duration, and follow-up period. When necessary, the study authors were contacted via email for clarification or additional data.

#### 2.1.5. Risk of Bias and Quality Assessment

Given that the available literature predominantly comprises case reports, the methodological quality of all the included studies was independently assessed by two authors (AT and ES) using the Joanna Briggs Institute (JBI) Critical Appraisal Checklists for Case Reports [71]. Each checklist consists of eight questions evaluating aspects such as patient demographics, clinical history, diagnostic procedures, interventions, outcomes, and potential sources of bias. Responses for each item were recorded as “Yes,” “No,” “Unclear,” or “Not Applicable.” Discrepancies between reviewers were resolved through discussion. Additionally, the two institutional cases presented in this review were subjected to the same quality assessment to ensure consistency across all the analyzed data. No formal meta-analysis was conducted due to the descriptive and heterogeneous nature of the data. Although a formal assessment of risk of bias due to missing results was not performed, we acknowledge the possibility of bias, particularly since several studies could not be retrieved despite extensive efforts. The certainty of the evidence was not formally assessed, reflecting the inherent limitations of case reports and case series.

#### 2.1.6. Data Synthesis

A narrative, descriptive synthesis was conducted. Data were summarized in tables and text. Continuous variables are presented as medians with 95% confidence intervals, while categorical variables are reported as frequencies and percentages. No quantitative pooling or effect estimation was performed due to heterogeneity and the case-based nature of the included reports.

### 2.2. Case Reports

This study retrospectively evaluated the clinical and histopathological characteristics of two patients who underwent simultaneous laparoscopic cholecystectomy and appendectomy for synchronous AC and AAP in our surgical department. The detailed documentation included preoperative imaging, laboratory data, intraoperative findings, surgical procedures, histopathological analyses, and postoperative outcomes. Since this investigation involved the retrospective analysis of anonymized patient data without intervention, local institutional ethical approval was not required in accordance with national regulations.

### 2.3. Statistical Analysis

Descriptive statistical analysis was performed using IBM SPSS Statistics for Windows, version 25.0 (IBM Corporation, Armonk, NY, USA). Continuous variables were presented as medians with 95% confidence intervals (CIs). Categorical variables were summarized as absolute numbers and percentages.

## 3. Results

### 3.1. Systematic Literature Analysis

**Figure 1:**
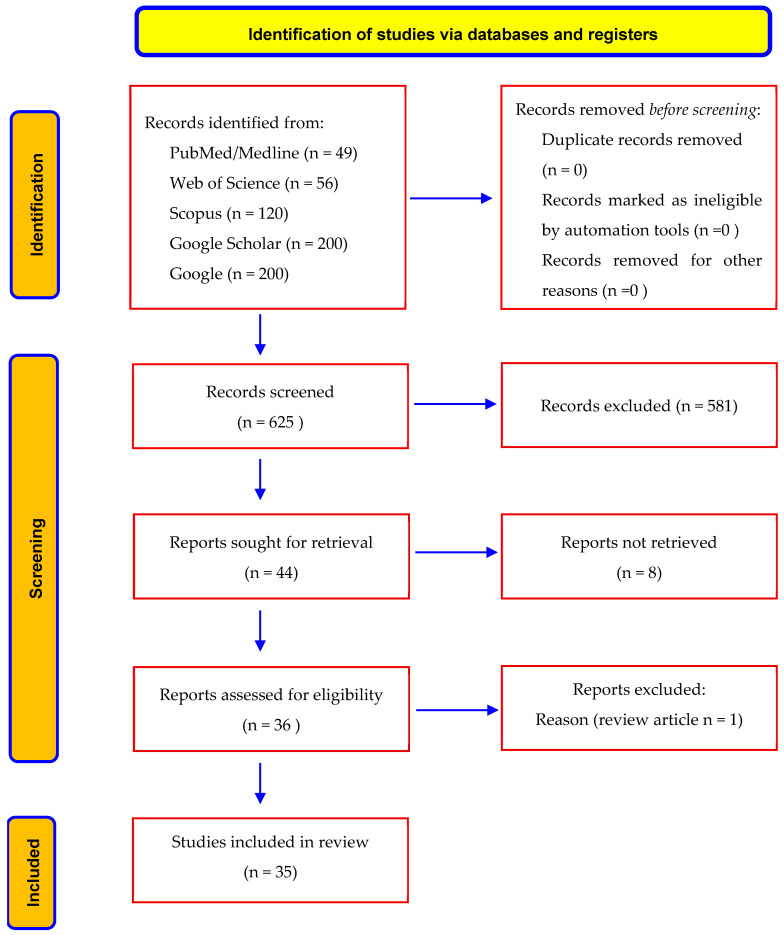
Prisma flowchart.

A total of 625 records were identified through database searching (PubMed: 49, Web of Science: 56, Scopus: 120, Google Scholar: 200, Google: 200). No duplicates were found, so all the records underwent title and abstract screening, resulting in the exclusion of 581 articles. Of the remaining articles, 44 reports were sought for eligibility assessment. Of the remaining 44 articles assessed for eligibility, one was excluded for lacking individual case data [30], and eight could not be retrieved due to language or accessibility barriers (seven in Russian, one in Spanish) [27,28,29,31,32,33,34,35]. Ultimately, 35 studies (34 in English, one in Slovak) comprising 38 patients were included in the analysis (Figure 1, Table 1 and Table 2) [5,15,19,36,37,38,39,40,41,42,43,44,45,46,47,48,49,50,51,52,53,54,55,56,57,58,59,60,61,62,63,64,65,66]. Additionally, two cases from our institution were incorporated, yielding a total of forty cases for qualitative synthesis.

In total, 40 cases were analyzed. The median age of the cohort was 41 years old (95% CI: 36–58), while the median WBC count was 13,950 (95% CI: 12,200–16,200). The cohort exhibited an equal gender distribution, with median ages of 50 years old (95% CI: 38–68) for males and 36 years old (95% CI: 32–58) for females. The median WBC count for males was 13,040 (95% CI: 12,170–15,390), whereas for females it was 16,000 (95% CI: 13,300–18,500). There were no statistically significant differences between genders in age (*p* = 0.223) or WBC count (*p* = 0.285). Complete blood counts were available for 33 patients, 25 of whom had leukocytosis.

Clinical presentation data were available for 37 of the 40 patients. All patients presented with abdominal pain of variable intensity, radiating to the right upper quadrant, epigastric region, or right lower quadrant. Sixteen patients experienced fever, another sixteen reported nausea, nineteen had vomiting, and five reported anorexia. Less common symptoms included constipation, diarrhea, chills, and hyperglycemia.

Diagnostic modalities were reported in thirty-seven cases, with fourteen undergoing both US and CT, nine only US, seven only CT, five diagnosed clinically, and two evaluated using US, CT, and MRI. Preoperative diagnosis data were available for all the patients. Twenty-four were diagnosed with synchronous AC and AAP, eleven with AC alone (including one case of a perforated gallbladder), four with AAP alone, and one underwent a diagnostic laparotomy.

Treatment modalities were documented for all patients. Laparoscopic cholecystectomy combined with appendectomy was performed in 27 patients. Open cholecystectomy combined with appendectomy was performed in eight patients. One patient underwent laparoscopic cholecystectomy and appendectomy that was converted to open surgery and required a small bowel resection. One patient was treated with percutaneous cholecystostomy with intravenous antibiotics alone. Another patient was managed conservatively with intravenous antibiotics. One patient underwent delayed laparoscopic cholecystectomy combined with open appendectomy. Finally, one patient initially underwent percutaneous cholecystostomy and later a delayed laparoscopic cholecystectomy, but refused appendectomy.

Postoperative complications were documented for 36 patients. Five developed significant complications, including sepsis with multiple organ dysfunction syndrome, sepsis with gastrointestinal perforation, ileostomy due to leakage, spontaneous abortion at 13 weeks of pregnancy, severe diarrhea, and wound infection.

Histopathological findings from appendectomy specimens were available for 34 patients. AAP was identified in twenty-five specimens, gangrenous appendicitis in four, perforated gangrenous appendicitis in one, perforated appendiceal diverticulitis in one, acute-on-chronic appendicitis in one, and a low-grade appendiceal mucinous neoplasm in one specimen. Histopathological examination of cholecystectomy specimens was performed in 32 cases. Fourteen specimens showed AC, seven had gangrenous cholecystitis, seven had acute-on-chronic cholecystitis, two had acalculous cholecystitis, one had low-grade biliary intraepithelial neoplasia with chronic cholecystitis, and one had perforated cholecystitis. The demographic and clinical characteristics of the forty patients, including the two from our institution, are summarized in Table 1 and Table 2.

### 3.2. Quality Assessment Results

The quality assessment using the JBI Critical Appraisal Checklist showed that most of the included case reports provided adequate detail in the key domains, such as patient demographics, clinical history, diagnostics, interventions, and outcomes. Out of 36 evaluated reports, including our institutional report, the majority were rated as high quality, with only a few showing unclear or incomplete reporting in some areas. This indicates that the data extracted for this systematic review is generally reliable and suitable for descriptive analysis. The results of the quality assessment are presented in Table 3.

### 3.3. Institutional Case Reports

#### 3.3.1. Case 1

**Figure 2:**
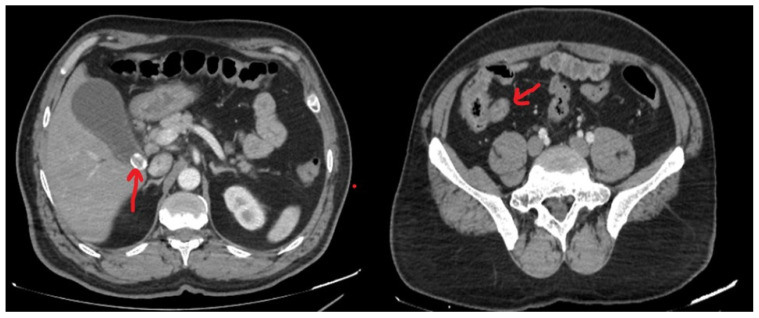
Axial computed tomography (CT) images of the same patient. The left image demonstrates a gallstone obstructing the cystic duct, gallbladder wall thickening, and a hydropic gallbladder. The right image reveals radiologic features consistent with AAP (The red arrow in the left image points to the gallstone located within the hydropic gallbladder, while the red arrow in the right image indicates the vermiform appendix).

A 52-year-old male presented to the emergency department with progressively worsening abdominal pain, nausea, and fever, which had begun three days prior. On admission, laboratory investigations revealed a hemoglobin level of 15.7 g/dL, white blood cell (WBC) count of 11.9 × 10^9^/L, C-reactive protein (CRP) of 6.2 mg/L, and blood glucose of 202 mg/dL. Physical examination demonstrated generalized abdominal tenderness, with a positive Murphy’s sign in the right upper quadrant, as well as guarding and rebound tenderness at McBurney’s point in the right lower quadrant. The patient’s medical history included hypertension, diabetes mellitus, and coronary artery disease. Abdominopelvic computed tomography (CT) revealed a hydropic gallbladder containing a 15 mm gallstone lodged at the neck and a gallbladder wall thickness of 5 mm. Additionally, pelvic imaging demonstrated an appendix with a diameter of 7 mm (Figure 2).

The patient underwent laparoscopic cholecystectomy and appendectomy. The cholecystectomy was performed using the standard American technique, followed by insertion of an additional 5 mm trocar in the left lumbar region to facilitate the appendectomy. The postoperative course was uneventful, and the patient was discharged on the third postoperative day.

**Figure 3:**
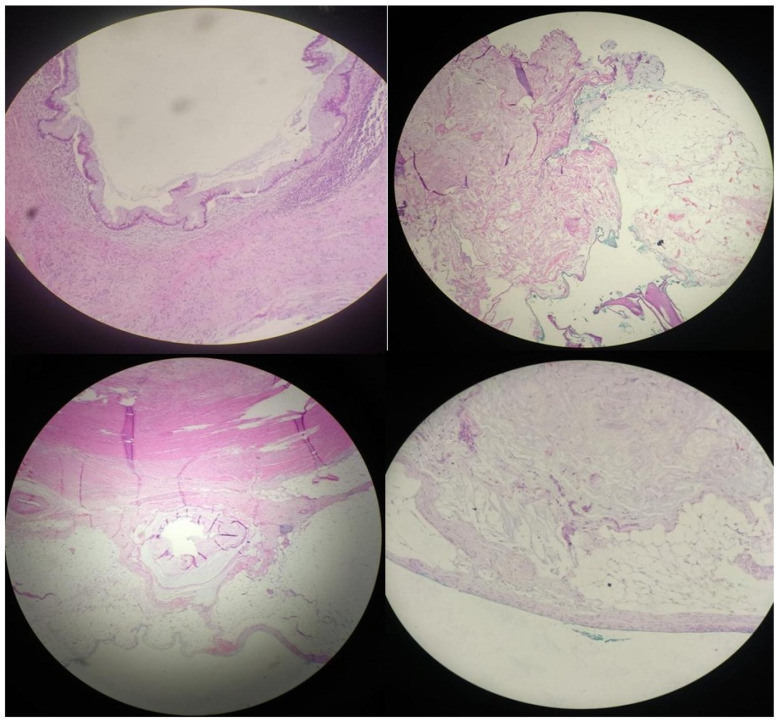
Histopathological images demonstrating biliary intraepithelial neoplasia (BilIN) in the gallbladder wall at various magnifications.

**Figure 4:**
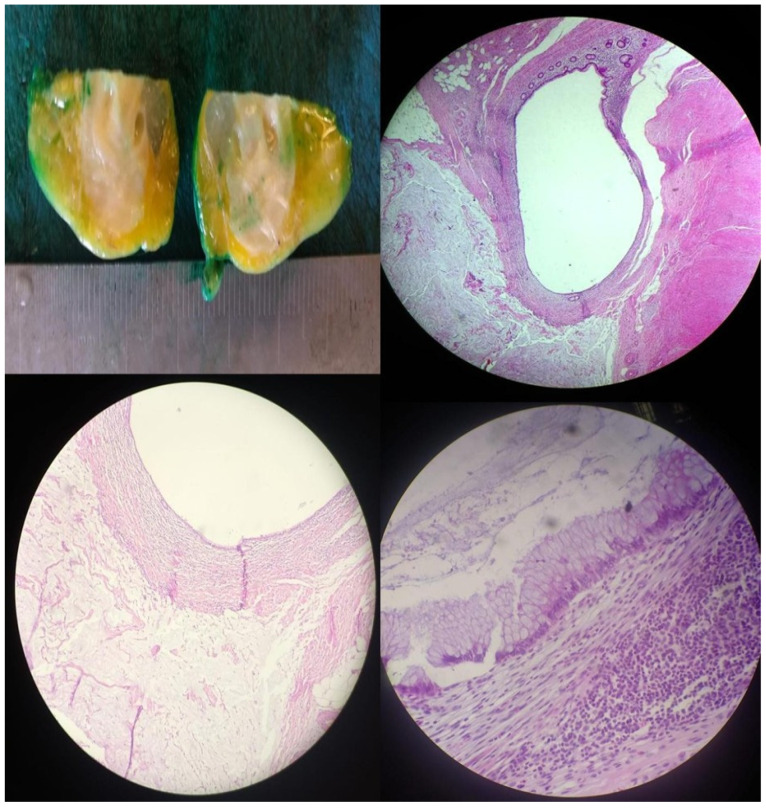
Histopathological images showing mucin pools in the distal muscularis propria and subserosa of the appendix. The muscularis propria displays low-grade mucinous neoplastic epithelium and mucin pools, visualized at various magnifications (4×, 10×, 40×; H&E staining). Low-grade mucinous neoplastic epithelium is also evident in the muscularis propria at higher magnification (40×, H&E).

Histopathological examination revealed chronic cholecystitis with low-grade biliary intraepithelial neoplasia (BilIN) (Figure 3). The appendectomy specimen demonstrated a low-grade appendiceal mucinous neoplasm (LAMN) (Figure 4), localized to the distal appendix and measuring 1.2 cm in maximum diameter. The tumor exhibited morphological features consistent with LAMN and showed infiltration into the muscularis propria and subserosa, with extension to the visceral peritoneum as acellular mucin pools. No evidence of lymphovascular or perineural invasion was identified. The tumor was staged as pT4a (Figure 4), and proximal surgical margins were clear. Follow-up 18F-fluorodeoxyglucose positron emission tomography/computed tomography (18F-FDG PET/CT) performed one month postoperatively demonstrated no residual disease. The patient remains disease-free after two years of oncological surveillance.

#### 3.3.2. Case 2

A 32-year-old female presented to the emergency department twice within two days due to persistent and worsening abdominal pain, accompanied by nausea and fever. Physical examination revealed tenderness and guarding in the right upper quadrant. Her medical history included a previous episode of acute calculous cholecystitis. On admission, laboratory investigations showed a hemoglobin level of 12.7 g/dL, white blood cell (WBC) count of 11.16 × 10^9^/L, C-reactive protein (CRP) level of 48.3 mg/L, aspartate aminotransferase (AST) level of 55 U/L, and alanine aminotransferase (ALT) level of 70 U/L. Contrast-enhanced abdominal computed tomography (CT) revealed a hydropic gallbladder containing numerous millimetric gallstones and a gallbladder wall thickness of 5 mm. Despite initial conservative management with oral antibiotics, proton pump inhibitors, and analgesics, the patient’s symptoms persisted, necessitating an emergency laparoscopic cholecystectomy.

**Figure 5:**
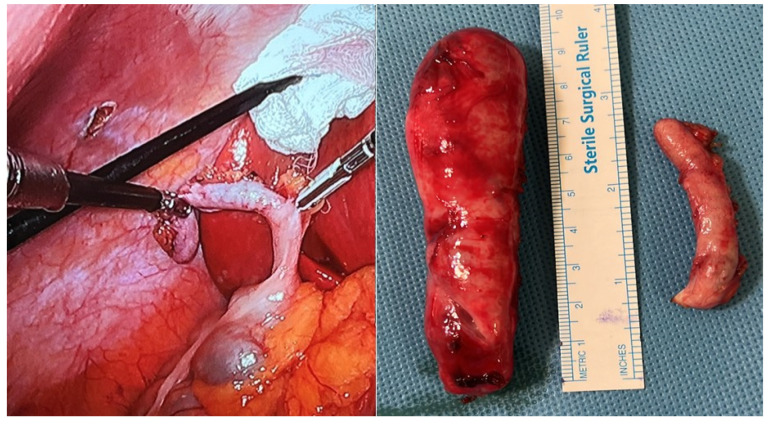
Left: intraoperative laparoscopic view during appendectomy. Right: gross specimens of the gallbladder and appendix resected during the same surgical procedure.

Intraoperative findings included a distended gallbladder with an edematous wall and minimal reactive fluid in the lower abdomen. Before proceeding with the cholecystectomy, the gallbladder was decompressed using a Veress needle to facilitate the procedure. Further inspection revealed an edematous and enlarged appendix located in the upper right quadrant beneath the liver. Given this unexpected finding, a laparoscopic appendectomy was performed utilizing the same trocar sites (Figure 5). The patient experienced an uneventful postoperative course and was discharged on the third postoperative day. Histopathological analysis confirmed gallstones and inflammatory changes in both the appendix and gallbladder specimens.

## 4. Discussion

The synchronous occurrence of AC and AAP is an exceptionally rare and diagnostically challenging condition, with only a limited number of reported cases in the international medical literature. This rarity highlights the lack of robust evidence to guide clinical decision making. In this discussion, we synthesize findings from 35 articles reporting 38 patients, along with two additional cases from our institution, focusing on the diagnostic challenges, underlying pathophysiological mechanisms, treatment strategies, clinical outcomes, and implications for best possible clinical practice.

One of the fundamental challenges in identifying synchronous AC and AAP is their overlapping clinical presentations [5]. Both conditions manifest with abdominal pain, fever, nausea, vomiting, and elevated inflammatory markers, sometimes accompanied by hyperbilirubinemia. However, the localization of pain often differs—right upper quadrant in AC and right lower quadrant in AAP—making differential diagnosis critical. Several studies have highlighted the importance of imaging modalities such as US and CT in resolving diagnostic ambiguities. CT, in particular, was noted for its ability to detect coexisting pathologies effectively when initial clinical examinations were inconclusive. However, in pregnant patients—where the use of CT is limited, especially for suspected AAP—diagnostic ambiguity remains extensively debated within the literature, particularly in the context of balancing maternal–fetal safety against the risks of delayed surgical management. The balance between the risks of a negative laparotomy to maternal and fetal health and the complications associated with delayed diagnosis represents a major consideration in clinical decision making. Therefore, pregnant patients may benefit from MRI examinations when available [72].

The exact pathophysiological mechanisms behind the concurrent presentation of AC and AAP remain unclear [39]. Three relevant mechanisms have been discussed in the literature [39,63]. The first involves pathogen predilection, referring to the tendency of certain bacteria to affect specific organs, as observed in historical experiments where bacteria cultured from human cases induced similar diseases in animal models. The second mechanism relates to portal venous bacteremia and endotoxemia, which have been shown in previous studies to cause cholestatic changes and liver dysfunction due to Gram-negative bacterial infections such as Escherichia coli, and may also contribute to sepsis-induced cholestasis. The third mechanism concerns increased bacterial translocation into the portal venous system as a result of intestinal mucosal ischemia, potentially leading to seeding of the liver and biliary tract, or invasion of the portal system from the vermiform appendix. In addition to these, systemic infections like Salmonella, as well as factors such as impaired immune regulation or biliary stasis, may also contribute to the development of synchronous inflammation in both the gallbladder and the vermiform appendix, highlighting how pathogen affinity, sepsis-induced cholestasis, and portal system invasion might converge to produce this rare clinical scenario [30,47,50,54,62,63,66].

Considering all these hypotheses, it can be hypothesized that inflammatory changes might result from the involvement of certain pathogens with a potential tendency to affect both the vermiform appendix and the gallbladder. Nonetheless, further investigations are required to support this hypothesis. Future studies should focus on examining specimens using advanced molecular microbiological techniques to identify and characterize the relevant bacterial strains. Such research may provide deeper insights into the underlying mechanisms of synchronous infections and facilitate the development of more specific diagnostic and therapeutic approaches.

Surgical intervention is typically required for the management of concurrent AC and AAP. Laparoscopic procedures are generally preferred over open surgery, though their success also depends on surgical team experience and available resources [5,41,54,64]. Laparoscopic procedures, as they are known to provide well-established advantages in performing either cholecystectomy or appendectomy individually, also offer similar benefits when both procedures are performed simultaneously. These benefits include reduced morbidity, shorter hospital stays, and quicker recovery periods [5]. This observation is supported both by our personal clinical experience and by data from relevant studies in the literature. Studies such as those by Nahidi et al. [55] and Fennelly et al. [46] have demonstrated that a four-port laparoscopic technique can effectively manage both conditions in a single surgical session, particularly when both pathologies are confirmed preoperatively. However, in patients with significant comorbidities or high surgical risk, conservative management remains an option, as shown in reports like that of O’Connor et al. [56], who described successful treatment with prolonged antibiotic therapy in an elderly patient.

The literature widely supports medical treatment followed by elective cholecystectomy as the standard approach for the management of AC [73]. While the popularity of non-operative management with antibiotics for uncomplicated AAP has recently increased, appendectomy remains the preferred treatment modality [12]. However, a non-operative approach with antibiotics may be more appropriate when synchronous AC and AAP occur in patients with significant comorbidities [62]. The treatment options may vary depending on the complexity of the conditions affecting the gallbladder and vermiform appendix. The current review highlights that the patient’s clinical status significantly influences the selection of surgical techniques. For instance, some patients undergo simultaneous laparoscopic cholecystectomy and appendectomy, while others require a combination of laparoscopic cholecystectomy and open appendectomy, simultaneous open surgeries, or a delayed cholecystectomy following an appendectomy [15,30,66].

The majority of patients undergoing timely surgical management for synchronous AC and AAP experience favorable clinical outcomes with minimal perioperative complications; however, delayed diagnosis or intervention can lead to severe outcomes, including perforation, generalized peritonitis, or severe infections [42,52,54,55]. Upon evaluation of the data from the forty patients included in this review, it was observed that three patients experienced serious postoperative complications, including sepsis, perforation, and multiple organ dysfunction syndrome [52,54,55]. In one case, a 70-year-old male with a history of colon cancer and cardiovascular disease initially developed AC, which was managed with percutaneous drainage. However, he subsequently developed sepsis and multiple organ dysfunction syndrome, and later was found to have AAP due to stone migration, though surgery was not performed and he improved with conservative management [52]. Another case involved a 68-year-old male with hypertension and chronic obstructive pulmonary disease who underwent surgery for AC. During the operation, a jejunal perforation and gangrenous appendicitis were discovered, and despite multiple surgical interventions, he died on the sixth postoperative day due to sepsis and multiple organ dysfunction syndrome [55]. The third case involved a 36-year-old woman who was 13 weeks pregnant. Initially diagnosed with gangrenous perforated appendicitis, she underwent an appendectomy. Shortly thereafter, she developed severe right upper quadrant pain, and imaging revealed gangrenous cholecystitis, prompting a second laparoscopic cholecystectomy. Postoperatively, she experienced a stump blowout at the appendectomy site, requiring a third surgery with a diverting loop ileostomy. Unfortunately, she suffered a spontaneous miscarriage during this period but ultimately recovered and was discharged [54]. These serious complications appear to be primarily related to significant underlying conditions affecting immune function in two patients, and to pregnancy in the third patient, where overlapping pregnancy symptoms may obscure diagnosis and clinical management tends to prioritize a conservative approach for fetal safety.

Pregnancy presents unique diagnostic and management challenges in cases of suspected concurrent AC and AAP. Physiological and anatomical changes during pregnancy can mask typical symptoms, delay diagnosis, and increase the risk of complications for both the mother and the fetus [74,75]. Imaging modalities such as US and MRI are preferred to minimize fetal radiation exposure [76]. Laparoscopy is generally safe during pregnancy, particularly in the second trimester, but requires careful monitoring [77]. Management decisions must balance maternal health with fetal safety, emphasizing timely surgical intervention to prevent severe outcomes.

Regardless of the initial preoperative diagnosis, performing a thorough exploration of the entire abdominal cavity during laparoscopy or laparotomy is crucial, as many reported cases in the literature have revealed unexpected synchronous pathologies that might otherwise be overlooked. This comprehensive approach ensures that coexisting diseases, such as concurrent AC and AAP, are promptly identified and appropriately treated, ultimately improving patient outcomes. Only two cases of synchronous appendicitis and cholecystitis in pregnant women have been reported in the literature. As previously described, Lew et al. [54] reported that a 13-week pregnant woman experienced severe complications including gangrenous appendicitis, subsequent gangrenous cholecystitis, and a stump blowout, ultimately resulting in a spontaneous miscarriage. In contrast, Grimes et al. [67] reported a 10-week pregnant patient who underwent surgery for perforated cholecystitis due to Salmonella paratyphi, experienced no complications, and carried her pregnancy to term. These contrasting cases highlight how pregnancy can both mask early symptoms and increase the risk of severe complications, emphasizing the need for prompt diagnosis and careful surgical management in pregnant patients with suspected intra-abdominal pathology.

This comprehensive analysis underscores the critical need for multidisciplinary diagnostic strategies, incorporating advanced imaging modalities, meticulous clinical evaluation, and prompt surgical consultation to optimize patient outcomes in cases presenting with ambiguous abdominal pain and potential concurrent intra-abdominal pathologies. Clinicians must remain vigilant for rare but severe concurrent conditions, particularly in patients with systemic infections or atypical presentations. The integration of advanced imaging technologies and laparoscopic techniques should be considered standard practice in addressing these cases.

Neoplasms of the appendix are rare and account for only about 0.5% of all gastrointestinal neoplasms. However, between 0.9% and 1.7% of appendectomy specimens contain a tumor, with approximately half of these identified as LAMNs, which are characterized by mucin production and slow progression [78]. Approximately 1% to 3.5% of cholecystectomy specimens reveal incidental BilIN [79]. BilIN represents a spectrum of precancerous epithelial changes in the gallbladder mucosa and is increasingly recognized as a potential precursor to gallbladder carcinoma. Its identification in routine histopathological examination underscores the importance of thorough tissue assessment, even in cases initially presumed to be benign. To our knowledge, aside from our current study, there are no reports in the literature documenting the simultaneous occurrence of LAMN and BilIN in the same patient. Moreover, it is crucial to emphasize that the early detection of these precancerous conditions and achieving complete resection through simple appendectomy and cholecystectomy may result in long-term survival benefits for the patient. Although evidence is limited, it remains unclear whether synchronous inflammatory processes in the appendix and gallbladder might predispose individuals to or unmask underlying neoplastic changes. This question directly relates to our observation of LAMN and BilIN coexisting in the same patient, suggesting that inflammation could potentially serve as a trigger or facilitating factor for tumorigenesis in these organs. Therefore, further studies are warranted not only to elucidate the potential pathophysiological mechanisms underlying this rare coexistence but also to explore any possible link between synchronous inflammation and the development of underlying neoplastic lesions.

This study has several inherent limitations. Firstly, the rarity of synchronous AC and AAP limits the generalizability of the findings and precludes definitive conclusions regarding its epidemiology, risk factors, and pathophysiology. Secondly, the retrospective nature of this systematic review introduces potential biases related to incomplete data reporting, variable diagnostic standards, and heterogeneous treatment protocols across the included case reports. Thirdly, the inability to retrieve eight studies, particularly those published in Russian and Spanish, may have resulted in selection bias and an incomplete representation of the global literature. Furthermore, inter-study variability in imaging modalities, surgical approaches, and histopathological evaluation methodologies further complicates cross-comparison. Finally, due to the limited sample size, larger multi-institutional prospective studies and international case registries are warranted to validate these observations and refine the diagnostic and therapeutic algorithms for this uncommon clinical condition.

## 5. Conclusions

Concurrent AC and AAP are rare but generally not particularly challenging to diagnose. Diagnosis is usually established through a combination of medical history, physical examination, and imaging modalities such as ultrasound or CT. Standard surgical principles generally apply. Rarely, laparoscopic procedures performed for one of these two uncommon conditions incidentally reveal synchronous inflammation in the other. Furthermore, our review demonstrates that performing both laparoscopic cholecystectomy and appendectomy concurrently is feasible and yields favorable outcomes, supporting its use as a safe and effective approach in suitable patients. These conclusions are supported by our systematic analysis of 40 cases, which revealed comparable clinical features, imaging findings, and positive outcomes following concurrent surgical management. Further studies are needed to clarify the underlying causes and mechanisms of this dual pathology, including possible infectious, immunological, or vascular factors. Moreover, the incidental detection of neoplastic lesions in some cases raises questions about a potential association with concurrent inflammation, highlighting the need for additional research. Routine histopathological examination of surgical specimens remains crucial, as it may reveal unexpected findings that significantly impact patient management and therapeutic decisions.

## Figures and Tables

**Table 1:** Summary of the main characteristics of published studies on synchronous AC and AAP.

References	Year	Lang.	Country	Age	Sex	Initial Presentation	Fever	Radiological Tools	WBC	Preoperative Diagnosis
Aljunaydil	2025	English	S.Arabia	30	F	Pain + Vomiting + Anorexia	Afebrile	CT + US	17.590	AAP + AC
Fennelly	2024	English	Australia	63	M	Pain + Vomiting	Afebrile	CT + US	28.100	AAP + AC
Ashfaq	2024	English	Pakistan	32	F	Pain + Nausea + Anorexia + Fever	37.7	US	Elevated	AAP + AC
Mahajan	2024	English	India	11	F	Pain + Vomiting	Febrile	US	14.000	AAP + AC
Błaszczyszyn	2024	English	Poland	66	F	Pain + Nausea	Afebrile	CT + US	17.600	AAP + AC
Flores	2023	English	Ecuador	77	M	Pain	Afebrile	US	13.040	AC
Kancheva	2023	English	USA	31	M	Pain + Nausea + Vomiting + Fever	38.4	CT + US	12.170	AAP + AC
Aceves-Ayala	2022	English	Mexico	14	M	Pain	Afebrile	US	12.500	AAP + AC
Alkhurmudi	2022	English	S.Arabia	36	F	Pain	Afebrile	US	7.540	AC
Nahidi	2021	English	USA	41	M	Pain + Vomiting + Fever + Chills	Febrile	CT + US	Normal	AAP + AC
Nahidi				68	M	Pain + Nausea + Vomiting	Afebrile	CT + US	NA	AC (Bowel Perforation)
Thompson	2021	English	UK	21	F	Pain + Nausea + Vomiting	37	Clinical	16.000	AAP
Thompson				50	M	Pain + Nausea + Vomiting	Afebrile	CT	12.200	AAP
Al-Nabulsi	2021	English	UK	62	M	Pain + Nausea + Vomiting	Afebrile	CT + US	13.900	AAP + AC
David	2021	English	UK	58	F	Pain	NA	CT + US	NA	AAP + AC
Lew	2021	English	USA	36	F	Pain	Afebrile	CT + US + MR	10.500	AAP
Lambe	2021	English	Ireland	70	M	Pain + Fever	Febrile	CT	NA	AAP + AC
Zhao	2020	English	China	90	M	Pain + Diarrhea	39.4	CT + US	15.390	AAP + AC
Joseph	2020	English	Australia	13	M	Pain + Diarrhea	Febrile	Clinical	8.000	AAP + AC
O’Connor	2020	English	UK	90	M	Pain	Febrile	CT	15.000	AAP + AC (Amyand hernia)
Alam	2019	English	Australia	15	F	Pain + Nausea + Vomiting	37	Clinical	26.000	AAP + AC
Sedik	2018	English	S.Arabia	35	F	Pain + Vomiting + Diarrhoea	37.7	CT + US + MRCP	54.000	AC
Jankovič	2018	Slovak	Slovakia	38	M	Pain + Nausea + Vomiting	Subfebrile	CT + US	16.200	AAP + AC
Victory	2017	English	USA	40	M	Pain + Nausea + Vomiting	39.2	CT + US	5.100	AAP + AC
Salih	2016	English	Iraq	66	F	Pain + Nausea + Fever	38.4	US	Normal	AC
Shweiki	2016	English	USA	29	F	Pain + Nausea + Vomiting	NA	CT	13.300	AAP + AC
Padron-Arredondo	2016	English	Mexico	43	F	Pain + Fever	38.5	US	16.200	AC
Gandhi	2015	English	N.Zealand	67	F	Pain + Nausea + Anorexia	Afebrile	CT	18.500	AAP + AC
Gattorno	2015	English	USA	41	M	Pain + Vomiting + Fever + Chills	Febrile	CT + US	Normal	AAP + AC
Lee	2014	English	Taiwan	78	M	Pain + Nausea	37.8	CT + US + ERCP	9.650	AAP + AC
DeMuro	2012	English	USA	45	F	Pain + Nausea + Vomiting	36.6	CT + US	8.000	AAP + AC
Sahebally	2011	English	Ireland	23	M	Pain + Nausea + Vomiting + Anorexia	38.5	US	14.300	AAP + AC
Pal	2011	English	S:Arabia	11	M	Pain + Vomiting + Fever	39	US	21.100	AAP + AC
Grimes	1996	English	USA	36	F	NA (Pregnancy 10 wk)	NA	NA	NA	AC
Rubin	1979	English	USA	55	F	Pain	37.2	Clinical	10.7	AC
Buzzard	1982	English	Australia	NA	NA	Pain	NA	NA	NA	AC
Buzzard				NA	NA	Pain	NA	NA	NA	AC
Black	1977	English	Australia	76	F	Pain	37.8	Clinical	19.000	AAP
Present Case			Turkey	52	M	Pain + Nausea + Fever	NA	CT	11.900	AAP + AC
Present Case				32	F	Pain + Nausea	NA	CT	11.160	AC

AAP: acute appendicitis; AC: acute cholecystitis; NA: non-available; CT: computed tomography; US: ultrasonograpy; MRCP: magnetic resonance cholangiopancreatography; ERCP: endoscopic retrograde cholangiopancreatography.

**Table 2:** Summary of the clinical characteristics of published studies on synchronous AC and AAP.

Management	Postoperative Complications	Histopathological Features (Appendix)	Histopathological Features (Gallbladder)	Discharge POD (Days)
Cholecystectomy + Appendectomy (Lap)	No	AAP	Acute on chronic cholecystitis	1
Cholecystectomy + Appendectomy (Lap)	No	Appendiceal diverticulitis (Perforated)	AC (Gangrenous)	3
Cholecystectomy + Appendectomy (Lap)	No	NA	NA	1
Cholecystectomy + Appendectomy (Lap)	No	AAP	AC	3
Cholecystectomy + Appendectomy (Lap)	No	NA	NA	10
Cholecystectomy + Appendectomy (Lap)	No	AAP (Gangrenous)	AC	2
Cholecystectomy + Appendectomy (Lap)	No	AAP (Gangrenous)	Acute on chronic cholecystitis	2
Cholecystectomy + Appendectomy (Lap)	No	AAP	AC	2
Cholecystectomy + Appendectomy (Lap)	No	AAP	AC	2
Cholecystectomy + Appendectomy (Lap)	No	AAP	AC (Gangrenous)	3
Cholecystectomy +Appendectomy (Converted to Open) + Small Bowel Resection	Sepsis + Perforation	AAP (Gangrenous)	AC (Gangrenous)	6 (Exitus)
Cholecystectomy + Appendectomy (Lap)	No	AAP	AC	2
Cholecystectomy + Appendectomy (Lap)	No	AAP	AC	2
Cholecystectomy + Appendectomy (Lap)	No	AAP	AC	2
Cholecystectomy + Appendectomy (Lap)	No	AAP	AC	NA
Cholecystectomy + Appendectomy (Lap)	Ileostomy (appendix complication)	AAP (Gangrenous)	Acute on chronic cholecystitis	NA (Fetal abortus)
Cholecystostomy (Percutan) + Antibiotics	Sepsis + MODS	No surgery	No surgery	60
Cholecystectomy + Appendectomy (Lap)	No	Acute on chronic appendicitis	AC (Gangrenous)	5
Cholecystectomy + Appendectomy (Lap)	Diarrhea	AAP	AC	7
Conservative treatment with IV antibiotics	No surgery	No surgery	No surgery	12
Cholecystectomy + Appendectomy (Lap)	No	AAP	AC (Gangrenous)	NA
Cholecystectomy + Appendectomy (Open)	No	AAP	AC	14
Cholecystectomy(Delayed-Lap) + Appendectomy (Open)	No	AAP (Gangrenous)	Chronic Cholecystitis	7
Cholecystectomy + Appendectomy (Lap)	No	AAP	Acute on chronic cholecystitis	2
Cholecystectomy + Appendectomy (Open)	No	NA (Sample lost)	AC	NA
Cholecystectomy + Appendectomy (Lap)	NA	AAP	AC	NA
Cholecystectomy + Appendectomy (Open)	No	AAP (Perforated)	AC	5
Cholecystectomy + Appendectomy (Lap)	No	AAP	AC	2
Cholecystectomy + Appendectomy (Lap)	No	AAP	Acute on chronic cholecystitis	3
Cholecystostomy (Percutan.) + Delayed Cholecystectomy (Lap)+ Refused appendectomy	No	NA (Appendectomy not performed)	NA	14
Cholecystectomy + Appendectomy (Lap)	No	AAP	Acute on chronic cholecystitis	2
Cholecystectomy + Appendectomy (Lap)	No	AAP	AC (Gangrenous)	2
Cholecystectomy + Appendectomy (Lap)	No	AAP	AC	3
Cholecystectomy + Appendectomy (Open)	No	AAP	AC (Perforated)	NA
Cholecystectomy + Appendectomy (Open)	No	AAP (Perforated)	AC (Gangrenous)	10
Cholecystectomy + Appendectomy (Open)	NA	AAP	AC	2
Cholecystectomy + Appendectomy (Open)	NA	AAP	AC	2
Cholecystectomy + Appendectomy (Open)	Wound infection	AAP	AC (Gangrenous)	18
Cholecystectomy + Appendectomy (Lap)	No	LAMN	Chronic Cholecystitis, Low Grade BilIN	3
Cholecystectomy + Appendectomy (Lap)	No	AAP	Acute on chronic cholecystitis	3

AAP: acute appendicitis; AC: acute cholecystitis; NA: non-available; POD: postoperative days; LAMN: low-grade appendiceal mucinous neoplasm; BilIN: biliary intraepithelial neoplasia; MODS: multiple organ dysfunction syndrome.

**Table 3:** The quality evaluation of 36 case reports (including our cases) was conducted using the JBI critical appraisal checklist for case reports.

References	Q1	Q2	Q3	Q4	Q5	Q6	Q7	Q8
Aljunaydil 2025 [39]	Y	Y	Y	Y	Y	Y	Y	Y
Fennelly 2024 [46]	Y	Y	Y	Y	Y	Y	Y	Y
Ashfaq 2024 [41]	Y	Y	Y	Y	Y	Y	U	Y
Mahajan 2024 [19]	Y	Y	Y	Y	Y	Y	Y	Y
Błaszczyszyn 2024 [5]	Y	Y	Y	Y	Y	Y	Y	Y
Flores 2023 [15]	Y	Y	Y	Y	Y	Y	Y	Y
Kancheva 2023 [51]	Y	Y	Y	Y	Y	Y	Y	Y
Aceves-Ayala 2022 [36]	Y	Y	Y	Y	Y	Y	Y	Y
Alkhurmudi 2022 [40]	Y	Y	Y	Y	Y	Y	Y	Y
Nahidi 2021 [55]	Y	Y	Y	Y	Y	Y	Y	Y
Thompson 2021 [64]	Y	Y	Y	Y	Y	Y	Y	Y
Al-Nabulsi 2021 [37]	Y	Y	Y	Y	Y	Y	Y	Y
David 2021 [44]	Y	N	Y	Y	Y	N	N	Y
Lew 2021 [54]	Y	Y	Y	Y	Y	Y	Y	Y
Lambe 2021 [52]	Y	Y	Y	Y	Y	Y	Y	Y
Zhao 2020 [66]	Y	Y	Y	Y	Y	Y	Y	Y
Joseph 2020 [50]	Y	Y	Y	Y	Y	Y	Y	Y
O’Connor 2020 [56]	Y	Y	Y	Y	Y	Y	Y	Y
Alam 2019 [38]	Y	Y	Y	Y	Y	Y	Y	Y
Sedik 2018 [62]	Y	Y	Y	Y	Y	Y	Y	Y
Jankovič 2018 [49]	Y	Y	Y	Y	Y	Y	Y	Y
Victory 2017 [65]	Y	Y	Y	Y	Y	Y	Y	Y
Salih 2016 [61]	Y	Y	Y	Y	Y	Y	Y	Y
Shweiki 2016 [63]	Y	Y	Y	Y	Y	N	N	Y
Padron-Arredondo 2016 [57]	Y	Y	Y	Y	Y	Y	Y	Y
Gandhi 2015 [47]	Y	Y	Y	Y	Y	Y	Y	Y
Gattorno 2015 [48]	Y	Y	N	N	Y	Y	Y	Y
Lee 2014 [53]	Y	Y	Y	Y	Y	Y	Y	Y
DeMuro 2012 [45]	Y	Y	Y	Y	Y	Y	Y	Y
Sahebally 2011 [60]	Y	Y	Y	Y	Y	Y	Y	Y
Pal 2011 [58]	Y	Y	Y	Y	Y	Y	Y	Y
Grimes 1996 [67]	Y	U	U	U	Y	Y	Y	U
Rubin 1979 [59]	Y	Y	Y	Y	Y	Y	Y	Y
Buzzard 1982 [43]	N	N	N	N	Y	N	N	Y
Black 1977 [42]	Y	Y	Y	Y	Y	Y	Y	Y
Present 2025	Y	Y	Y	Y	Y	Y	Y	Y

Y: Yes, N: No, U: Unclear.

## Data Availability

The datasets analyzed during the current study are available from the corresponding author on reasonable request.
